# Can voriconazole gradient diffusion testing results be extrapolated to isavuconazole and posaconazole in *Aspergillus* spp.? Comparative analysis with CLSI broth microdilution and *cyp51A* gene sequencing

**DOI:** 10.1128/aac.01813-25

**Published:** 2026-05-15

**Authors:** Roya Vahedi-Shahandashti, Anna-Sophia Nickel, David Eisele, Cornelia Lass-Flörl, on behalf of ISHAM Working Group Member of Intrinsic Antifungal Resistance

**Affiliations:** 1 European Confederation of Medical Mycology (ECMM) Excellence Center, Institute of Hygiene and Medical Microbiology, Medical University of Innsbruck 27280, Innsbruck, Austria; University Children's Hospital Münster, Münster, Germany

**Keywords:** surrogate marker, Etest, azole resistance, *cyp51* gene, voriconazole, antifungal susceptibility testing

## Abstract

This study assessed whether voriconazole (VRC) gradient diffusion results can be extrapolated to isavuconazole (ISA) and posaconazole (PSC) in *Aspergillus fumigatus* and *Aspergillus terreus* by comparison with the Clinical and Laboratory Standards Institute (CLSI) broth microdilution (BMD) and *cyp51A* sequencing. Agreement with CLSI BMD was highest at 48 h, but remained limited for some azole-species combinations. VRC-PSC extrapolation was reliable in both species, whereas VRC-ISA extrapolation was species-dependent. Gradient diffusion underestimated resistance detection in some *cyp51A*-mutant isolates, highlighting the risk of false-susceptible interpretations.

## INTRODUCTION

*Aspergillus* species are abundant opportunistic molds that cause invasive aspergillosis (IA) in immunocompromised hosts (1). *Aspergillus fumigatus* is the leading cause of IA and is associated with high mortality (2, 3), followed by *Aspergillus terreus*, which is predominant in some centers and intrinsically resistant to amphotericin B (4, 5). Azoles, particularly voriconazole (VRC) and isavuconazole (ISA), are recommended first-line treatment for aspergillosis (6, 7), but rising azole resistance and breakthrough infections complicate management (8–11). Most azole resistance is linked to mutations in the *cyp51A* gene, which encodes the 14α-sterol demethylase (9, 12), making antifungal susceptibility testing (AFST) increasingly important, especially in suspected azole resistance, treatment failure, or for surveillance (6). Since VRC is the key first-line agent (6, 13), it is often the only azole tested in susceptibility testing panels, and its minimum inhibitory concentration (MIC) result is extrapolated to other azoles. Validating such extrapolation across different AFST methods, including Clinical and Laboratory Standards Institute (CLSI) and European Committee on Antimicrobial Susceptibility Testing (EUCAST) broth microdilution (BMD) and gradient diffusion testing, which is widely used in routine diagnostic laboratories due to its faster and simpler workflow, is essential (14, 15).

This study evaluated the reliability of VRC gradient diffusion test as a surrogate marker for ISA and posaconazole (PSC) susceptibility and compared gradient diffusion strip-derived MICs with CLSI BMD and *cyp51A* sequencing. *A. fumigatus* (*n* = 11) and *A. terreus* (*n* = 9) isolates were selected to include predominantly azole-resistant/non-wild-type (non-WT) isolates and a smaller proportion of susceptible/wild-type (WT) isolates. All *A. fumigatus* isolates were identified by partial β-tubulin sequencing in the present study, as described previously (8), whereas *A. terreus* isolates included in this study had been molecularly identified either by partial β-tubulin sequencing (8) or by random amplified polymorphic DNA (RAPD) method (16) in prior investigations and were, therefore, not re-sequenced/re-analyzed in the current work.

Susceptibility testing was performed by applying two methods: CLSI BMD in-house-prepared panels according to the CLSI M38 guideline (17) and gradient diffusion method, utilizing VRC (bioMérieux, Lot number: 1010651670), PSC (bioMérieux, lot number 1010922220), and ISA (Liofilchem, lot number 121224084) strips, with each antifungal from the same lot number to remove inter-lot variability, according to the manufacturer’s instructions and prior description (18, 19). Briefly, isolates were cultured on Sabouraud agar medium (bioMérieux; Vienna, Austria) for 3–5 days for *A. fumigatus* and *A. terreus*, respectively. Spores were harvested from one originating Sabouraud agar plate culture using sterile water containing 0.1% Tween 20 (Sigma-Aldrich, Lot SLCB2671) and subsequently filtered using a cell strainer (pluriSelect pluriStrainer, 40 μm, Lot V-P94-2025-09, Germany), and the resulting suspension was used for both CLSI BMD and gradient diffusion methods. For the CLSI broth microdilution (BMD) method, spores were counted using a Neubauer chamber, and the inoculum was prepared according to CLSI BMD guideline (17). For the gradient diffusion method, the inoculum density was adjusted to a 0.5 McFarland standard (approximately 1 × 10⁶ spores/mL) based on optical density using sterile 0.85% NaCl, as recommended by the manufacturer, and evenly inoculated onto ready-to-use RPMI 1640 agar plates (Axon-Lab, Tyrol, Austria). After allowing the surface to dry (approximately 15 min), gradient diffusion strips were applied, and plates were incubated at 37°C. For each isolate, the same standardized inoculum was used for susceptibility testing of the three azoles. All tests were performed in duplicate using biological replicates utilizing independent fresh spore suspensions prepared from separate subcultures of the same isolate on Sabouraud agar medium. If the MIC values differed within ±2 dilutions, the higher value was chosen; and if the results differed by more than two dilutions, the test was repeated. CLSI BMD MICs were read at 48 h (17), whereas for gradient diffusion testing, manufacturers primarily recommend an initial MIC reading at 24 h, with an additional reading at 48 h used for confirmation or when growth at 24 h was insufficient for reliable interpretation (20, 21). Gradient diffusion strip-derived MICs rounded up to the nearest higher twofold dilution for comparison with CLSI BMD twofold dilution scheme. Essential agreement (EA) was defined as MICs within ±2 dilutions and categorical agreement (CA) as identical interpretive categories based on CLSI BMD clinical breakpoints (CBPs) or epidemiological cutoff values (ECVs) (22–24). Very major error (VME) was defined as the percentage of isolates classified as susceptible by the gradient diffusion method but resistant by the reference CLSI BMD, representing false-susceptible results. *Cyp51A* mutations were sequenced and analyzed exclusively for *A. fumigatus* isolates in this study as described previously (25, 26) using the reference genome GCF_000002655. In contrast, for *A. terreus* isolates, mutation or wild-type status was obtained from earlier investigations (8, 16); accordingly, the present study relied on these existing data without repeating the analyses.

Table 1 summarizes the susceptibility profiles of *A. fumigatus* and *A. terreus* to VRC, ISA, and PSC together with associated *Cyp51A* alterations. Across all azoles, the CLSI BMD method yielded higher MICs than the gradient diffusion method, consistent with previous studies (15, 27–29). Gradient diffusion MICs showed a clear time-dependent shift, with lower values at 24 h and increased MICs at 48 h, indicating that reading time substantially influences method comparability (Table 1). CA between gradient diffusion readings and CLSI BMD at 48 h generally improved when gradient diffusion method results were read at 48 h rather than 24 h, although the extent of improvement varied by species and azole (Table 2). For VRC, CA increased markedly at 48 h in *A. fumigatus* (90.9%), whereas only limited improvement was observed for *A. terreus* (55.5%). ISA exhibited improved CA at 48 h in both species, but without meeting the ≥90% acceptance criterion. For PSC, CA could not be assessed in *A. fumigatus* due to the absence of CBPs or ECVs, while *A. terreus* demonstrated only a modest increase at 48 h (89%) compared to 24 h (85.7%) (Table 2). Overall, these findings indicate that later gradient diffusion readings improve categorical alignment with CLSI BMD for some, but not all, species–azole combinations. In line with this, VME rates in *A. fumigatus* were higher at 24 h (25% for VRC and 37.5% for ISA) and decreased at 48 h (12.5% for both azoles), while VME analysis was not performed for *A. terreus* due to the absence of established clinical breakpoints.

**TABLE 1:** Minimum inhibitory concentrations (MICs) of voriconazole (VRC), isavuconazole (ISA), and posaconazole (PSC) for *A. fumigatus* and *A. terreus* isolates determined by the gradient diffusion (24 and 48 h) and CLSI broth microdilution (BMD) (48 h) methods, along with corresponding *cyp51A* alterations and changes in susceptibility categorization according to the CLSI (22–24).

Isolates	Antifungals	AFST method/MIC (µg/mL)			*cyp51A* alteration	Categorization^*a*^		
		CLSI BMD	Gradient diffusion			CLSI BMD	Gradient diffusion	
			24 h	48 h			24 h	48 h
*A. fumigatus* (A19)	VRC	4	0.125	0.25	M220V	R	S	S
	ISA	8	0.5	0.5		R	S	S
	PSC	1	0.5	1		NA^*b*^	NA	NA
*A. fumigatus* (A68)	VRC	8	1	4	TR34/L98H	R	I	R
	ISA	>16	2	>32		R	I	R
	PSC	2	2	4		NA	NA	NA
*A. fumigatus* (A71)	VRC	16	2	4	TR34/L98H	R	R	R
	ISA	16	8	>32		R	R	R
	PSC	1	2	4		NA	NA	NA
*A. fumigatus* (A20)	VRC	8	2	8	TR34/L98H	R	R	R
	ISA	>16	2	>32		R	I	R
	PSC	2	4	4		NA	NA	NA
*A. fumigatus* (A116)	VRC	8	1	2	TR34/L98H	R	I	R
	ISA	16	2	4		R	I	R
	PSC	2	2	4		NA	NA	NA
*A. fumigatus* (A142)	VRC	4	0.5	2	M263I, D345G	R	S	R
	ISA	4	1	2		R	S	I
	PSC	1	0.5	2		NA	NA	NA
*A. fumigatus* (A145)	VRC	8	1	2	TR34/L98H	R	I	R
	ISA	16	1	4		R	S	R
	PSC	1	1	2		NA	NA	NA
*A. fumigatus* (1098)	VRC	0.5	0.06	0.06	WT	S	S	S
	ISA	1	0.25	0.25		S	S	S
	PSC	0.25	0.06	0.125		NA	NA	NA
*A. fumigatus* (A141)	VRC	16	2	8	TR34/L98H	R	R	R
	ISA	>16	4	>32		R	R	R
	PSC	2	4	8		NA	NA	NA
*A. fumigatus* (3031)	VRC	0.5	0.06	0.06	WT	S	S	S
	ISA	1	0.25	0.25		S	S	S
	PSC	1	0.06	0.125		NA	NA	NA
*A. fumigatus* (7236)	VRC	0.5	0.125	0.125	WT	S	S	S
	ISA	1	0.25	0.25		S	S	S
	PSC	0.5	0.125	0.25		NA	NA	NA
*A. terreus s.s.* (16)	VRC	4	1	2	WT (8)	Non-WT	WT	WT
	ISA	2	0.5	1		Non-WT	WT	WT
	PSC	1	0.25	0.5		WT	WT	WT
*A. terreus s.s.* (X12)	VRC	2	0.25	2	M217V (16)	WT	WT	WT
	ISA	4	0.25	4		Non-WT	WT	Non-WT
	PSC	1	0.06	1		WT	WT	WT
*A. terreus s.s.* (X14)	VRC	2	0.5	1	WT (16)	WT	WT	WT
	ISA	4	1	2		Non-WT	WT	Non-WT
	PSC	1	0.125	1		WT	WT	WT
*A. terreus s.s.* (X15)	VRC	8	1	1	WT (16)	Non-WT	WT	WT
	ISA	8	1	2		Non-WT	WT	Non-WT
	PSC	1	0.25	0.5		WT	WT	WT
*A. terreus s.s.* (X19)	VRC	2	0.25	0.5	M217V (16)	WT	WT	WT
	ISA	4	1	2		Non-WT	WT	Non-WT
	PSC	2	0.06	0.5		Non-WT	WT	WT
*A. terreus s.s.* (X24)	VRC	2	0.5	0.5	M217V (16)	WT	WT	WT
	ISA	4	0.5	2		Non-WT	WT	Non-WT
	PSC	0.5	0.125	1		WT	WT	WT
*A. terreus s.s.* (X23)	VRC	4	0.5	2	M217V (16)	Non-WT	WT	WT
	ISA	4	1	4		Non-WT	WT	Non-WT
	PSC	1	0.06	0.5		WT	WT	WT
*A. terreus s.s.* (X21)	VRC	4	0.5	1	M217V (16)	Non-WT	WT	WT
	ISA	8	–^*c*^	2		Non-WT	WT	Non-WT
	PSC	1	–	1		WT	–	WT
*A. terreus s.s.* (T68)	VRC	0.5	–	0.25	WT (8)	WT	–	WT
	ISA	1	–	0.25		WT	–	WT
	PSC	0.25	–	0.125		WT	–	WT

^*a*^ The categorization of both CLSI BMD and gradient diffusion methods, was based on the available CLSI clinical breakpoints (CBPs) and epidemiological cutoff values (ECVs).

^*b*^ NA, the absence of CBPs and ECVs for PSC and *A. fumigatus*.

^*c*^ –, growth was insufficient to obtain a readable result at that specific time point. Non-WT, Non-wild type; WT, wild type.

**TABLE 2:** Essential and categorical agreements between gradient diffusion and CLSI broth microdilution (BMD) results of voriconazole (VRC), isavuconazole (ISA), and posaconazole (PSC) at different reading time points for gradient diffusion testing method^*a*^

Species	Antifungal	Essential agreements %		Categorical agreements %	
		Gradient diffusion (24 h) vs. CLSI BMD (48 h)	Gradient diffusion (48 h) vs. CLSI BMD (48 h)	Gradient diffusion (24 h) vs. CLSI BMD (48 h)	Gradient diffusion (48 h) vs. CLSI BMD (48 h)
*A. fumigatus* (*n* = 11)	VRC	27.2	72.7	54.5	90.9
	ISA	45.4	90.9	45.4	81.8
	PSC	90.9	90.9	–^*b*^	–
*A. terreus* (*n* = 9)	VRC	37.5	87.5	50	55.5
	ISA	57.1	100	0	89
	PSC	42.8	100	85.7	89
All (*n* = 20)	VRC	32	80	53	75
	ISA	50	95	27.7	85
	PSC	72.2	95	–	–

^*a*^ EA, essential agreement (±2 dilution); CA, categorical agreement according to CLSI clinical breakpoints or epidemiological cutoff values (ECVs).

^*b*^ –, no clinical breakpoint or epidemiological cutoff value (ECV) was available for the respective antifungal-species combination.

Comparisons of CA across azoles highlighted the time dependence of gradient diffusion readings (Table 3). In *A. fumigatus*, CA between VRC and ISA increased from 81.8% at 24 h to 90.9% at 48 h by gradient diffusion method and reached 100% by CLSI BMD at 48 h (Table 3). In contrast, *A. terreus* showed pronounced variability, with high CA at 24 h (100%) but a marked decline at 48 h (22.2%) and poor agreement by CLSI BMD at 48 h (33.3%). When considering CLSI BMD data alone, VRC showed excellent EA with ISA for both species, consistent with a previous study (30). Consistent with CA, EA generally improved at 48 h in both species, with high agreement for ISA and PSC (95%) but only sub-threshold improvement for VRC (Table 2).

**TABLE 3:** Categorical agreement for voriconazole (VRC) and isavuconazole (ISA) across CLSI BMD and gradient diffusion testing methods at different reading times

Species	Categorical agreements %		
	VRC gradient diffusion vs. ISA gradient diffusion/24 h	VRC gradient diffusion vs. ISA gradient diffusion/48 h	VRC CLSI BMD/48 h vs. ISA CLSI BMD/48 h
*A. fumigatus* (*n* = 11)	81.8	90.9	100
*A. terreus* (*n* = 9)	100	22.2	33.3

**Fig 1:**
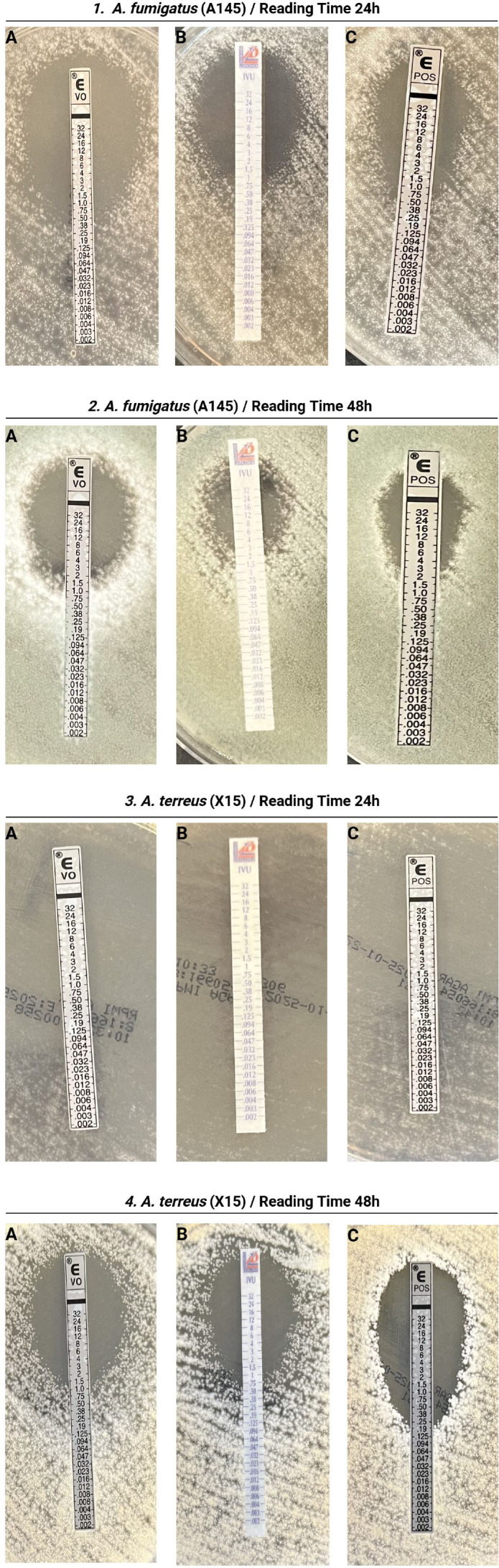
Representative gradient diffusion inhibition patterns of *Aspergillus* species at different reading time points. Panels show gradient strips for voriconazole (VRC) (**A**), isavuconazole (ISA) (**B**), and posaconazole (PSC) (**C**) placed on RPMI 1640 agar. Representative examples include: (1) *Aspergillus fumigatus* at 24 h reading time point; (2) *A. fumigatus* at 48 h reading time point; (3) *Aspergillus terreus* at 24 h reading time point; and (4) *A. terreus* at 48 h reading time point. The figure illustrates differences in inhibition ellipse between species and incubation times.

Fig. 1 provides representative examples of gradient diffusion testing inhibition patterns for *A. fumigatus* and *A. terreus* at 24 and 48 h, illustrating differences in growth and MIC readability across incubation times. Although our findings confirm that reading time affects gradient diffusion susceptibility method results, data on the impact of incubation duration on its performance for *Aspergillus* spp. remain limited and are largely inferred from yeast studies (15). The observed reading-time effects are consistent with the flexible nature of the gradient diffusion method, in which MIC interpretation depends on fungal growth characteristics rather than a single fixed endpoint (20, 21). Manufacturer recommendations emphasize an initial reading at 16–24 h with confirmation at 48 h, particularly for slower-growing molds (20, 21). Our data illustrate how this growth-dependent variability can influence categorical outcomes, highlighting the need to identify the most reliable reading time for each species-drug pair in routine diagnostics.

To evaluate which susceptibility testing method and reading time better reflect the underlying genetic background, results were analyzed in relation to *cyp51A* mutations. Among *A. fumigatus* isolates, three with wild-type *cyp51A* were susceptible to ISA and VRC by both CLSI BMD and gradient diffusion testing, whereas all eight carrying *cyp51A* substitutions (M220V, TR34/L98H, M263I, D345G) (26, 27) were resistant to VRC and ISA by CLSI BMD. Based on gradient diffusion 48 h readings, seven of these eight isolates were classified as VRC resistant and one as susceptible, whereas for ISA, gradient diffusion testing classified one isolate as susceptible, six as resistant, and one as intermediate (Table 1). Among *A. terreus* isolates, all five carrying the *cyp51A* mutation M217V (16) were classified as non-WT for ISA by both CLSI BMD and gradient diffusion at 48 h (Table 1). Two isolates were non-WT for VRC by CLSI BMD but remained WT by gradient diffusion testing at both reading times, and one isolate was non-WT for PSC by CLSI BMD while testing WT by gradient diffusion testing at 24 and 48 h (Table 1). Overall, CLSI BMD demonstrated superior detection of *cyp51A*-associated azole resistance compared with gradient diffusion testing, which underestimated resistance in some mutant isolates, highlighting the risk of false-susceptible interpretations when relying on gradient strip methods alone.

Limitations include the single-center design and the small sample size, which is an intentionally enriched set of 20 isolates with mostly resistant/non-WT, potentially precluding any inference on resistance prevalence. We focused on *A. fumigatus* and *A. terreus* and on specific commercial gradient strip assays, which may limit generalizability to other *Aspergillus* species, azoles, or testing platforms. Genotypic analysis was restricted to *cyp51A*, the common azole resistance determinant, and did not assess other potential resistance mechanisms (including alterations in *cyp51B*), and no clinical outcome data were available to correlate *in vitro* susceptibility findings with patient-level outcomes.

Despite the limited number of isolates, this study reveals low EA and CA for several species-azole combinations when gradient diffusion testing is compared with CLSI BMD, particularly at 24 h readings, underscoring important limitations of this method in routine diagnostics. Although 48 h gradient diffusion readings improved agreement, it remained suboptimal for key azoles, notably VRC and ISA, and resistance detection was underestimated in several *cyp51A*-mutant isolates at both time points, indicating a persistent risk of false-susceptible classification. While 48 h VRC results can be reliably extrapolated to PSC in both *A. fumigatus* and *A. terreus*, extrapolation to ISA is species- and time-dependent and should be applied cautiously, with direct ISA testing remaining advisable for *A. fumigatus* when resistance is suspected. Overall, 48 h gradient diffusion readings should be preferred while using this method, but CLSI BMD remains superior for detecting genetically mediated resistance, highlighting the need for larger multicenter studies to refine incubation times and extrapolation strategies.

## Data Availability

All data supporting the findings of this study are included in the article. The cyp51A sequences of *Aspergillus fumigatus* isolates (accession numbers PX619764–PX619774), as well as the corresponding β-tubulin sequences (accession numbers PX920950–PX920960), are publicly available.
